# Pencil Beam Scanning Proton Therapy for Adolescents and Young Adults with Head and Neck Sarcomas

**DOI:** 10.14338/IJPT-23-00010.1

**Published:** 2023-10-25

**Authors:** Miriam Vázquez, Katja Baust, Amaia Ilundain, Dominic Leiser, Barbara Bachtiary, Alessia Pica, Ulrike L. Kliebsch, Gabriele Calaminus, Damien C. Weber

**Affiliations:** 1 Center for Proton Therapy, Paul Scherrer Institute, ETH Domain, Villigen, Switzerland; 2 Department of Paediatric Haematology and Oncology, University Hospital Bonn, Bonn, Germany; 3 Department of Radiation Oncology, Inselspital, Bern University Hospital, University of Bern, Bern, Switzerland; 4 Department of Radiation Oncology, University Hospital of Zürich, Zürich, Switzerland

**Keywords:** adolescents, young adults, AYA, sarcoma, head and neck, proton therapy, pencil beam scanning, QoL

## Abstract

**Purpose:**

To assess clinical outcomes of adolescents and young adults (AYAs) with head and neck sarcomas (HNSs) treated with pencil beam scanning proton therapy (PBSPT) and to report quality of life (QoL).

**Materials and Methods:**

Twenty-eight AYAs (aged 15 to 39 years) with HNS treated between January 2001 and July 2022 at our institution were included. The median age was 21.6 years. Rhabdomyosarcoma (39.3%), Ewing sarcoma (17.9%), chondrosarcoma (14.3%), and osteosarcoma (14.3%) were the most frequent diagnoses. Three (10.7%) patients were metastatic before PBSPT and 13 (46.4%) patients had a tumor with intracranial extension. The median total radiation dose was 63 GyRBE (range, 45 to 74 GyRBE). Thirteen (46.4%) patients received concomitant chemotherapy. Toxicity was reported according to the Common Terminology Criteria for Adverse Events (CTCAE), version 5.0 (US National Institutes of Health, Bethesda, Maryland). Survival was estimated using the Kaplan-Meier method. QoL was assessed using a PEDQOL (Pediatric Quality of Life Questionnaire) questionnaire. Self-reported outcomes were assessed using institutional questionnaires.

**Results:**

With a median follow-up of 57 months (range, 3.7 to 243 months), 5 patients (17.8%) had local failure (LF) only, 2 (7.1%) experienced distant failure (DF) only, and 2 (7.1%) had LF and DF. The estimated 5-year local control (LC) and distant control (DC) rates were 71.8% and 80.5%, respectively. The median times to LF and DF were 13.4 and 22.2 months, respectively. Four (14.3%) patients died, all but one from their HNS. Estimated 5-year overall survival was 90.7%. Six (21.4%) patients developed nonocular grade ≥3 toxicity, which consisted of otitis media (n = 2), hearing impairment (n = 2), osteoradionecrosis (n = 1), and sinusitis (n = 1). Four (14.3%) patients developed cataracts that required surgery. The 5-year freedom from nonocular grade 3 toxicity was 91.1%. No grade 4 or higher toxicity was observed. Adolescents rated their quality of life before treatment worse than their parents did.

**Conclusion:**

Excellent outcomes with acceptable late-toxicity rates were observed for AYAs with HNS after PBSPT.

## Introduction

Adolescents and young adults (AYAs), usually aged between 12 and 24 or 39 years, constitute a distinctive patient population that faces unique challenges [1]. Sarcomas are a rare group of tumors that account for an overall incidence in Europe of 5.6 per 100,000 per year [2] and more commonly develop in AYAs [3, 4]. Fewer than 10% of sarcomas develop in the head and neck region [2], of which rhabdomyosarcoma (RMS), synovial sarcoma, Ewing sarcoma (EWS), and osteosarcoma are the most frequent types in this young population [4, 5]. Head and neck sarcomas (HNSs) usually require a multimodal approach that includes chemotherapy, surgery, and/or radiation. Surgical resection with adequate margins is usually challenging due to the proximity to several critical organs and the infiltrative nature of sarcomas. Radiation therapy is an often-required treatment modality that can improve local control (LC) in HNS but usually at the cost of important severe late toxicity [6]. Still, AYA patients with HNS face a 5-year relative survival rate of about 65% [2], which is also lower than that for children [7].

Proton beam therapy (PT) has been shown to be advantageous in sparing multiple organs at risk in head and neck tumors while maintaining or improving LC [8, 9]. Pencil beam scanning PT (PBSPT) is a highly conformal technique that allows for a more conformal delivery of radiation thanks to steeper dose distributions [10]. This beam delivery modality that avoids unnecessary exposure to radiation is especially relevant when treating AYAs, who face a substantial psychosocial and long-term symptom burden of disease [11]. To date, reports on PT for HNS have focused mainly on children, showing promising outcomes in terms of LC and toxicity [12–14]. Emerging research on carbon ion therapy (CIT) has demonstrated encouraging early results in adults with HNS, with acceptable levels of toxicity [15–17] and a 3-year LC of 72% to 92% [16, 17]. Moreover, particle therapy may allow for a dose escalation, leading to an increased LC while minimizing long-term toxicity, when treating radioresistant tumors such as sarcomas [16]. However, the majority of these reports are based on non-AYA cohorts and accounts with short follow-up periods [15–17].

As long survival after PT is expected, the assessment of health-reported quality of life (QoL) adds additional value to better understand the patient’s experience after the treatment [18, 19]. This is particularly relevant for AYAs in whom the tumor and its treatment may lead to disruptions in their physical, social, and emotional development. Here, we report clinical outcomes, toxicity, and self-reported QoL in AYAs after PBSPT for HNS.

## Materials and Methods

### Patient Characteristics

**Figure 1.**
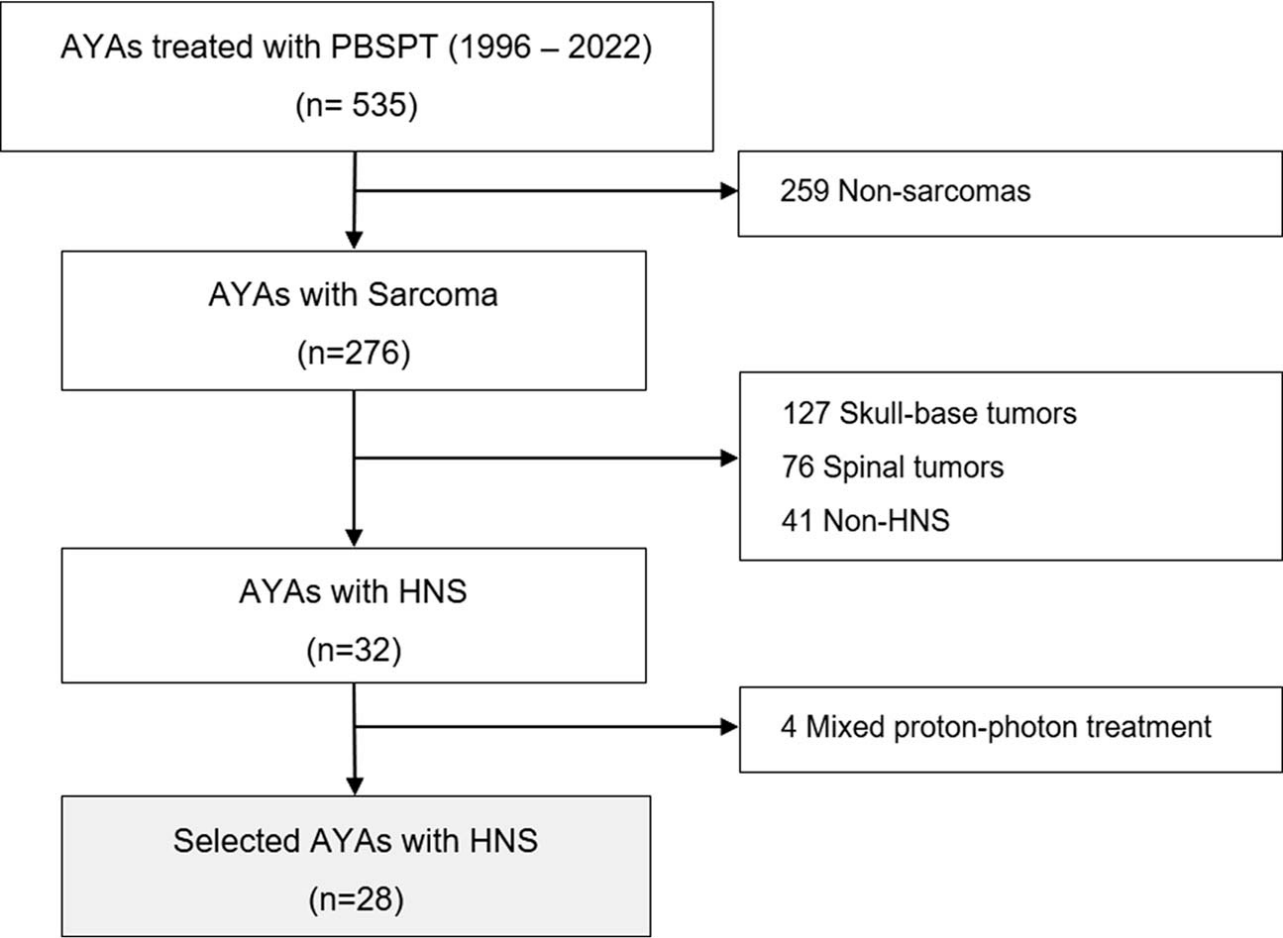
Patient selection flowchart.

Between January 2001 and July 2022, 535 AYAs were treated with PBSPT at our institution, of whom 276 (51.6%) had a diagnosis of a sarcoma. We excluded skull base tumors (n = 127), spine tumors (n = 76), and other non-HNSs (n = 41). Of the 32 remaining patients with HNS, 4 patients who received mixed proton-photon treatment were also excluded. A total of 28 patients were thus eligible for the analysis (Figure 1). The study was approved by the regional ethical review board (EKNZ 2021-02045).

Demographic data (age and sex) and tumor (histology, molecular characteristics, tumor site, tumor size, and stage), surgical (type of resection, resection margins, timing of the surgery, and number of surgeries), and chemotherapy (type of chemotherapy, systemic agents, and timing) characteristics were collected from our electronic medical records. The patients’ primary tumor, lymph node, and metastasis status were staged according to the 8th Classification of Malignant Tumors (TNM) of the American Joint Committee on Cancer [20]. Intracranial extension (ICE) diagnosis was based on radiological imaging as the growth of the tumor into the intracranial compartment with infiltration into or contact with the adjacent structures (bones, meninges, vessels, or brain tissue).

### Radiation Treatment Characteristics

Patients were treated using PBSPT at the scanning gantry with energy-degraded beams from the 590-MeV cyclotron until 2005 and the 250-MeV cyclotron after 2005. The proton dose calculation was computed using a three-dimensional dose calculation algorithm developed at the Paul Scherrer Institute. All patients were immobilized using a bite block or a thermoplastic mask.

The initial primary tumor at diagnosis was identified by gross tumor volume (GTV). An additional margin of 0.5 to 20 mm (median, 10 mm) was added to create the clinical target volume (CTV), which was also adapted to anatomical boundaries and shifts. Twenty-two (78.6%) patients received a boost to treat residual disease. The boost CTV (CTV_boost_) included the tumor bed or gross residual tumor volume (GTV_residual_) manually adapted to include areas at risk for potential residual disease. An additional margin of 4 to 7 mm (median, 5 mm) was given to CTV and CTV_boost_ to create the planning target volume (PTV) and the boost planning target volume (PTV_boost_) margin. Only one patient with a soft tissue sarcoma of the neck had a 7-mm margin to the PTV. Lymph node irradiation of the involved lymph nodes was performed on 3 (10.7%) patients.

Treatment plans were optimized using a single-field optimization or a multiple-field optimization method. The dose was prescribed to the mean in proton doses and expressed in terms of Gy in its relative biological effectiveness (GyRBE = proton Gy × 1.1) [21]. Organs at risk were contoured by the treating radiation oncologist according to consensus guidelines [22]. Dose-volume constraints were applied as stated by the protocol. Treatment was delivered in a median of 2 series.

### Follow-up and Events

The medical oncology or the radiation oncology team monitored patients every 3 to 6 months after the end of the PT. Follow-up consultations were performed at our institution or the referring institution for patients living far away. Acute and late toxicities were defined as the observed effects before and after 90 days following the completion of PT, respectively. Acute toxicity was assessed weekly during the treatment and after the completion of the treatment using the Common Terminology Criteria for Adverse Events (CTCAE), version 5.0 (US National Institutes of Health, Bethesda, Maryland). Data regarding the status of the patient, late toxicity, and recurrence were collected from the follow-up medical reports. Recurrences were evaluated by a radiation oncologist who reviewed the radiological images of recurrence and fused them with our performed treatment plan stored in our Paul Scherrer Institute planning system. In-field, marginal, and out-of-field local failures (LFs) were defined as recurrent disease within the 95% isodose, within the 50 to 94% isodose, and below the 50% isodose, respectively. Distant failure (DF) was defined as disease beyond the regional lymphatic area. Time to an event was defined as the time from the start of radiation therapy to failure or death.

### Quality of Life and Self-Reported Outcomes

Health-related QoL was investigated in an ethics-approved collaborative project on children that started in 2005 with the University of Bonn (EKNZ 2014-244). Quality of life was assessed using the PEDQOL, which is a questionnaire that includes eight domains (autonomy, emotional functioning, body image, cognition, physical functioning, social functioning—peers, social functioning—family, and subjective well-being). It was available in a proxy-rating version for the parents (PEDQOL-proxy) and a self-rating version for children aged between 5 and 17 years (PEDQOL-self) in various validated languages. After obtaining informed consent, the questionnaire was distributed before the start of PT (E1), 2 months after the end of PT (E2), and yearly thereafter (E3, E4, E5, etc). The calculated QoL score per domain ranges from 0 to 100, and higher QoL scores suggest better patient QoL [18]. For 9 patients between 15 and 17 years of age, a completed baseline (E1) proxy and/or self-rating questionnaire was available, for which descriptive values at E1 are presented for both self-reports and proxy reports (patients and parents) using box plots.

As part of our institutional follow-up, each patient was provided with an annual questionnaire addressing various aspects such as tumor status, treatment sequelae, social interactions, academic or professional performance, and medication use. A distinct questionnaire tailored to adolescents (**Supplemental Material S1**) was administered to patients below 18 years of age, whereas adult patients above 18 years of age received a separate version (**Supplemental Material S2**). A total of 14 (50%) patients completed at least one questionnaire. For those patients completing more than one questionnaire, negative answers (ie, reporting an impairment) in the last questionnaire were chosen. This study presents the qualitative findings gleaned from the responses to these questionnaires.

### Endpoints and Statistical Analysis

The primary endpoints of the analysis were overall survival (OS), LC, and distant control (DC). Overall survival was calculated from the date of the first day of PT until the date of death from any cause or was censored at the last follow-up. LC or DC was calculated from the date of the first day of PT until the date of LF or the date of DF, respectively. Secondary endpoints were toxicities (acute and late) and QoL. Actuarial local and distant failures and OS were analyzed by the Kaplan-Meier method. Statistical analysis was performed using IBM Corp. Released 2021. IBM SPSS Statistics for Windows, Version 28.0. Armonk, NY: IBM Corp.

## Results

### Patient and Treatment Characteristics

Twenty-eight AYAs were treated with PBSPT for HNS during the study period. Table 1 shows the demographic and clinical characteristics of the patients. The median age was 21.6 years (range, 15 to 37.9 years). Twelve (48.9%) patients were between 15 and <18 years of age. The most frequent primary diagnosis was RMS (39.3%) followed by EWS (17.9%). Seventeen (60.7%) patients had a soft tissue tumor, and 11 (39.3%) had a bone tumor. Soft tissue tumors were more frequently located in the nasal cavity/nasopharynx (n = 4; 23.5%) and infratemporal fossa (n = 4; 23.5%), while bone tumors arose in their majority from the ethmoid bone (n = 4; 36.4%). Fourteen (50%) patients had a grade 3 tumor (Table 1). Most (n = 17; 60.7%) patients had a T1 stage, 3 (10.7%) had involved lymph nodes, and 3 (10.7%) were metastatic. Thirteen (46.4%) patients had a tumor with ICE (Table 1), which consisted of 6 RMSs (46.1%), 3 EWSs (23.1%), 2 chondrosarcomas (15.4%), 1 osteosarcoma (7.7%), and 1 synovial sarcoma (7.7%). Four (14.3%) patients had a recurrent tumor, and 1 (3.5%) had a radiation-induced fibrosarcoma after a prior irradiation for a nasopharyngeal carcinoma. Among the 11 patients with RMS, 6 (54.5%) were embryonal, 4 (36.4%) were alveolar, and 1 (9.1%) was of the spindle cell subtype. FOXO1-PAX3 rearrangement was available and positive in 2 (50%) out of 4 patients with alveolar RMS.

**Table 1.** Demographic and clinical characteristics of AYAs with HNS.

Parameter	Value for all patients (n = 28)
Median age (years) (range)	23.7 (15-37.9)
Sex [no. (%)]	
Female	14 (50)
Male	14 (50)
Diagnosis [no. (%)]	
Rhabdomyosarcoma	11 (39.3)
Ewing sarcoma	5 (17.9)
Osteosarcoma	4 (14.3)
Chondrosarcoma	4 (14.3)
Synovial sarcoma	3 (10.7)
Fibrosarcoma	1 (3.5)
Tumor site [no. (%)]	
Soft tissue tumors (n = 17)	
Infratemporal fossa	4 (14.3)
Nasopharynx/nasal cavity	4 (14.3)
Paranasal sinus	2 (7)
Pterygoid fossa	1 (3.5)
Masticatory space	1 (3.5)
Oropharynx	1 (3.5)
Orbit	2 (7)
Neck	2 (7)
Bone tumors (n = 11)	
Maxilla	3 (10.7)
Ethmoid	4 (14.3)
Nasal	1 (3.5)
Frontal	1 (3.5)
Zygomatic	1 (3.5)
Mandible	1 (3.5)
Median tumor size (mm) (range)	42.5 (12-95)
Tumor histological grade [no. (%)]	
G1	1 (3.6)
G2	3 (10.7)
G3	14 (50.0)
GX	10 (35.7)
TNM [no. (%)]	
T	
T1	17 (60.7)
T2	4 (14.3)
T3	3 (10.7)
T4	4 (14.3)
N	
N1	3 (10.7)
N0	25 (89.3)
M	
M1	3 (10.7)
M0	25 (89.3)
Intracranial extension [no. (%)]	
Yes	13 (46.4)
No	15 (53.6)
Tumor status at PT [no. (%)]	
Newly diagnosed	24 (85.7)
Recurrent	4 (14.3)

**Abbreviations**: AYAs, adolescents and young adults; HNS, head and neck sarcoma; PT, proton therapy.

Table 2 shows the treatment characteristics. Twenty (71.4%) patients had a tumor surgical resection, while 8 (28.6%) had a biopsy only. Complete tumor resection was performed on 4 (14.3%) patients. Treatment according to a protocol was performed on 21 (75%) of the patients, and 1 (3.6%) was enrolled in a clinical trial. The list of protocols is detailed in **Supplemental Table S1**. Induction chemotherapy was given to the majority of patients (n = 20; 71.4%), and 13 (46.4%) received concomitant chemotherapy. Alkylating agents were the most frequently used chemotherapy agents (n = 20; 71.4%). The median radiation dose was 63 GyRBE (range, 45 to 74 GyRBE) in 1.8 GyRBE (range, 1.8 to 2.2 GyRBE) per fraction (Table 2).

**Table 2.** Treatment characteristics.

Parameter	Value for all patients (n = 28)
Margin surgery [no. (%)]	
R0 resection	4 (14.3)
R1 resection	12 (42.9)
R2 resection	4 (14.3)
Biopsy only	8 (28.6)
Use of chemotherapy [no. (%)]	
Induction	
Yes	20 (71.4)
No	8 (28.6)
Concomitant	
Yes	13 (46.4)
No	15 (53.6)
Maintenance	
Yes	15 (53.6)
No	13 (46.4)
Treatment inside a clinical protocol [no. (%)]	
Yes	1 (3.6)
No	27 (96.4)
Chemotherapy agents (no.)	
Alkylators	
Yes	20
No	8
Anthracyclines	
Yes	10
No	18
Platinum	
Yes	10
No	18
Radiation dose (GyRBE)	
Median total dose (range)	63 (45–74)
Median boost dose (range)	9 (0–20)
Median dose per fraction (range)	1.8 (1.8–2.2)

### Clinical Outcomes

After a median follow-up of 57 months (range, 37 to 243 months), 5 patients (17.9%) had LF only, 2 (7.1%) experienced DF only, and 2 (7.1%) had both. The estimated actuarial 5-year LC was 71.8% (95% confidence interval [CI], 49.4% to 85.5%) (**Supplemental Figure S1A**). The median time to LF was 13.4 months (range, 3.3 to 21.2 months). The estimated actuarial 5-year DC was 80.5% (95% CI, 55.2% to 92.4%) (**Supplemental Figure S1B**). The median time to DF was 22.2 months (range, 2.7 to 56.4 months). Four (14.3%) patients died, all but one as a result of the progression recurrence of their HNS. One patient developed metastatic breast cancer and died due to the disease. The estimated 5-year OS was 90.7% (95% CI, 67% to 97.6%) (**Supplemental Figure S1C**).

Patterns of failures are described in Table 3. Five patients developed only LF; all of them were in field. DF only was observed in 2 patients. Two patients developed both DF and LF. **Supplemental Tables S2**, **S3**, and **S4** show the clinical and treatment characteristics of patients with LF only, DF only, and both, respectively.

**Table 3.** Sites of local and distant failures.

Type(s) of failure	Site(s)	No. (%)
LF only (n = 5)	In field	5 (100)
	Marginal	0
	Out of field	0
DF only (n = 2)	Bone, meningeal, and distant lymph nodes	1 (50)
	Bone	1 (50)
LF and DF (n = 2)	Out-of-field LF/lung and distant lymph node metastases	1 (50)
	In-field/soft tissue metastases	1 (50)

**Abbreviations**: LF, local failure; DF, distant failure.

All patients developed acute toxicity. Six (21.4%) had only grade 1 toxicity, and 22 (78.6%) had grade 2 or higher. Common grade 2 acute toxicities included radiation dermatitis (46.4%) and mucositis (28.6%). Seven (25%) patients developed grade 3 toxicity, which consisted of 5 cases of mucositis and 2 cases of radiation dermatitis. One patient experienced both.

Nonocular late toxicity developed in 21 (75%) patients. Of these, 9 (32.1%) had only grade 1 toxicity, and 12 (42.8%) had grade 2 or higher toxicity. Overall, late hearing impairment grade ≥2 was observed in 4 (7.1%) patients. A grade 2 endocrinology disorder requiring hormonal replacement was observed in 2 (7.1%) patients. One (3.6%) case of brain radiation necrosis grade 1 was observed. Six (21.4%) patients developed nonocular grade ≥3 toxicity, which consisted of otitis media (n = 2), hearing impairment (n = 2), sinusitis (n = 1), and osteoradionecrosis (n = 1). Four (14.3%) patients developed cataracts that required surgery, and 1 (3.6%) developed retinopathy with a decrease in visual acuity. The actuarial 3-year freedom from nonocular grade 3 late toxicity was 91.6% (95% CI, 69.9% to 97.9%) (**Supplemental Figure S2**). The median time to nonocular grade 3 toxicity was 66.1 months (range, 3.4 to 161.9 months). No grade 4 or higher toxicity was observed.

### Quality of Life and Self-Reported Outcomes

**Figure 2.**
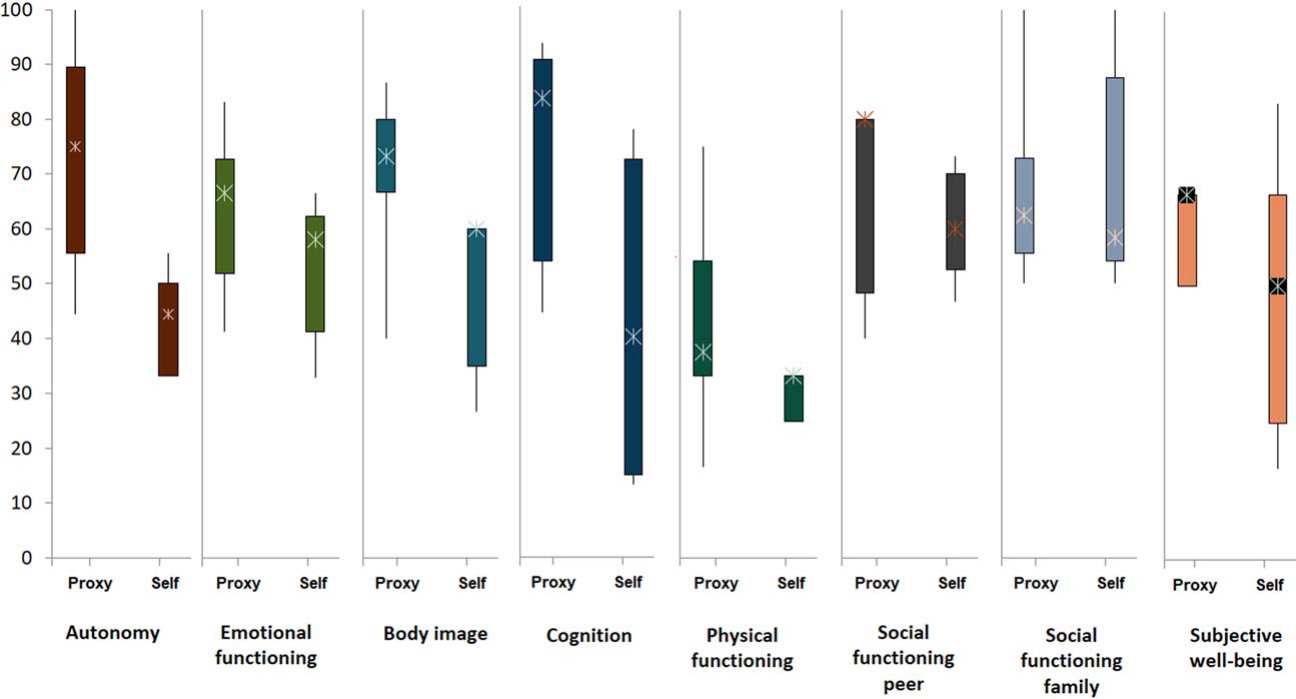
Proxy and self-assessments of QoL in adolescents treated with PBSPT at E1 (before proton therapy). Abbreviations: QoL, quality of life; PBSPT, pencil beam scanning proton therapy.

Eight parents completed the E1 proxy questionnaire, of whom 5 adolescent offspring completed the corresponding E1 self-rating questionnaire. Additionally, for one adolescent patient, only the baseline (E1) self-questionnaire was completed. In total, baseline QoL ratings (E1) before the start of the PT were available for a total of 9 patients. It was observed that adolescents consistently reported low scores in their self-ratings in all domains compared to those of parents in the proxy rating. The most pronounced difference in scores emerged in the domains of autonomy and cognition, while the differences were smaller for physical functioning and social interactions within the family (Figure 2). The clinical characteristics of this group of patients are shown in **Supplemental Table S5**.

**Figure 3.**
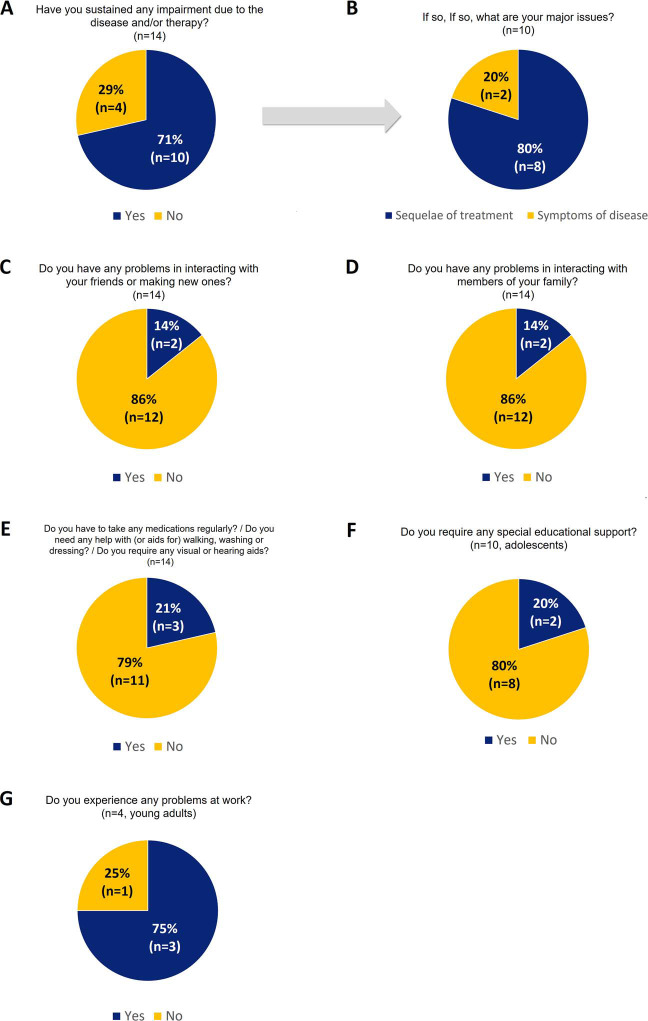
Self-reported outcomes on the impact of disease and/or therapy on various aspects of daily life and functioning.

A total of 14 patients (50%) completed at least one institutional follow-up questionnaire. The median time to the completion of the last questionnaire was 40.5 months (range, 3.1 to 243 months). Out of these patients, 10 (71.4%) indicated that the disease had adversely impacted their daily life (Figure 3A), with 8 attributing this to treatment sequelae (surgery or PT) and 2 attributing this to ongoing disease symptoms (Figure 3B). In terms of social interactions, 2 experienced difficulties with their friend group (Figure 3C) and also encountered issues with family members (Figure 3D). Treatment sequelae were cited as the cause of these impairments in social interactions. Regarding additional chronic therapies, 3 patients (21.4%) were utilizing chronic medication or special aid (Figure 3E), including physical therapy for trismus, hormone replacement therapy, hearing aids, and opioid medication for chronic pain management. Among adolescents (n = 10), 2 required additional educational support (Figure 3F) and reported a decline in academic performance. Of the 4 patients aged 18 years or older, 3 experienced work-related difficulties (Figure 3G).

## Discussion

This study presents an analysis of clinical and self-reported/proxy outcomes for an AYA cohort with HNS treated with PBSPT. Our 5-year LC, DC, and OS rates of 71.8%, 80.5%, and 90.7%, respectively, are in line with those of previous studies investigating adult patients with HNS treated with particle therapy [15–17]. We found that 75% of patients experienced some degree of late toxicity, underscoring the vulnerability of AYAs to radiation therapy in the head and neck region. Nevertheless, grade 3 nonocular late toxicity occurred in 21.4% of patients, which is comparable to recent reports of CIT in adults with HNS [16, 17]. A remarkable 5-year freedom from nonocular grade 3 toxicity of 91.6% supports the treatment’s safety. Interestingly, the adolescents rated their QoL before treatment worse than their parents did.

Among adults with unresectable HNS, 3-year LC and OS rates of 73% to 92% and 74% to 78% after treatment with PT or CIT have been reported [16, 17]. One of the potential advantages of particle therapy for HNS is the capacity to escalate the total radiation dose, which consequently may enhance the prognosis for these patients. A study performed at the Research Center for Charged Particle Therapy in Chiba, Japan, investigated the role of dose escalation among a cohort of patients with unresectable HNS, yielding encouraging results. They compared 27 patients receiving 70.4 GyE in 16 fractions of CIT or PT (n = 27) to a historical cohort of patients treated with 56.7 or 64 Gy of photon therapy delivered in a conventional fractionation regimen (n = 14). Those treated with 70.4 GyE showed improved 3-year LC (92% versus 23.6%) and a trend toward better 3-year OS (74.1% versus 42.9%) [16]. Likewise, Yang et al reported early 2-year local progression-free survival of 90% after a median follow-up of 15 months among 51 patients with unresectable HNS treated with CIT (80%) or PT (20%). The 2-year DF-free survival rate was only 57% [15], indicating the aggressive nature of these tumors, even when achieving good LC. Our 5-year LC of 71.7% is lower than those in the above-mentioned studies. One of the reasons for this might be the inclusion of patients who were reirradiated (n = 3) or had recurrent tumors (n = 3). Yang et al reported that those patients who received reirradiation exhibited worse 1-year local relapse-free survival (44% versus 81%) [15], which suggests that salvage treatment is unlikely to be curative after LF [23]. Furthermore, half of our patients had a high-grade tumor, and only 4 had an R0 resection, which are known risk prognosis factors for LF [12]. Additionally, nearly half of the patients presented with ICE, which was found to be correlated with a higher risk of LF among children with RMS [12, 24]. Our median times to LF and DF were 13.4 and 22.2 months, respectively. Most of the relapsed tumors consisted of LFs (n = 7, including 2 combined with DF). A recent study on the use of mixed proton and CIT in a small cohort (n = 15) of AYAs (age range, 14 to 32 years) with RMS showed similar results. With a median follow-up of 21 months, they reported times to LF and DF of 16.6 months and 9.2 months, respectively. Three patients developed LF, 4 developed DF, and 2 had both [25]. Nonetheless, comparisons between our findings and those from the previous studies are challenging given the shorter follow-up duration and the inclusion of adult participants across all age groups, while the presence of AYA individuals is unintentional. Even so, when considering the data from the European RARECARE registry, our 5-year OS of 90.7% appears favorable compared to the 5-year relative OSs of 65% for soft tissue HNS and 62% for all bone sarcomas [2]. However, further analysis and adjustments would be required to make a more accurate comparison to the data from this registry.

Our 5-year freedom from nonocular grade 3 toxicity of 91.6% is encouraging. Severe nonocular late toxicity occurred in 21.4% of cases, which is comparable to that in other HNS cohorts with rates of grade 3 or higher ranging from 19% to 80% [6, 16, 17]. Although Yang et al [15] reported excellent severe toxicity rates of only 2% after CIT or PT, we believe that this may be partly due to a shorter follow-up time (median, 15.7 months). In our study, the median time to grade 3 or higher late toxicity was over 5 years, highlighting the significance of these side effects in long-term survivors. Hearing impairment was observed in 4 patients (14.2%), with 2 cases being grade 3. In a study with a shorter follow-up period, Andrä et al found that 2 (7.1%) HNS patients treated with photon therapy developed grade 2 hearing impairment [6]. In our cohort, all hearing impairment cases were deemed unavoidable due to the tumor’s proximity to the cochlea. Infections requiring intravenous antibiotics or surgical intervention were seen in 3 patients (10.7%), which is higher and more severe than those in other studies on particle therapy for children with HNS [14, 24]. Consistently, infections constitute one of the major noncancer causes of death in AYAs [26]. Only one patient developed grade 3 bone radiation necrosis, in contrast to Jingu et al, who observed 4 cases (14.8%) after CIT [16]. Grade 3 ocular toxicity consisted mainly of cataracts, which could be easily corrected after lens replacement surgery. While 3.7% to 10% of HNS cases develop a grade ≥3 radiation-induced optic neuropathy [16, 17], we have not observed any case in our study. It is important to note that the above-mentioned studies are based on a small number and heterogeneous group of patients, which makes a comparison difficult. During the study period, one of our patients developed metastatic breast cancer and died due to the disease, but none of them had a radiation-induced second malignancy. AYA survivors with sarcomas experience significantly higher long-term mortality rates due to second cancers than the general population [26]. While PT has been suggested to reduce the estimated incidence of secondary tumors compared to conventional radiation therapy [27], this has not yet been confirmed [28]. The theorized advantage of PT may be evident only after PBSPT as it results in a lower total body dose due to neutrons [28]. Further follow-up is required to support this hypothesis.

AYA patients have been shown to exhibit poorer survival than children. A large epidemiological study (EUROCARE-5) showed that AYAs with osteosarcoma, EWS, and RMS experienced significantly lower 5-year relative survival than children, which sank between 5 and 28.8% depending on the tumor type [29]. Factors postulated to contribute to this phenomenon include biological aspects, diagnosis delays, different standard-of-care policies, and variations in access to specialized institutions or clinical trials [30]. In our study, only one patient was treated in a clinical trial. This might reflect a known issue. AYAs with sarcomas are less likely to be enrolled in pediatric treatment protocols [30]. The European Pediatric Soft Tissue Sarcoma Study Group (EpSSG) trial is an example of this, in which AYAs with RMS and other soft tissue sarcomas had a significantly lower rate of enrollment than children (50% versus 77% and 18% versus 64%) [31]. Moreover, AYAs with sarcomas face an additional barrier to access to PT. Bishop et al observed that insurance approval rates for this population are lower than those for children (67% versus 100%), and more than half of AYAs initially receive a denial for coverage, which may result in prolonged waiting times for authorization, leading to potential treatment delays and financial strain [32].

An essential aspect to consider when treating AYA patients with HNS is the impact of treatment on their QoL. We observed that attending adolescents reported lower scores in all domains than their parents did (Figure 2). Even if considering the low number of self-report ratings, this is unusual compared to previous reports on QoL among children, who scored significantly better than their parents [33]. One possible explanation for this finding could be that teenagers, who are at a stage of increased self-awareness and sensitivity, might perceive the impact of their condition more acutely than their parents, especially in view of their growing need for autonomy. For the self-reported outcomes on follow-up institutional questionnaires, the majority of the surveyed patients (71.4%) reported that the disease or treatments negatively impacted their daily lives. Social interactions, requirements for educational support, or difficulties at work were also pointed issues. Lim et al reported a higher unemployment rate after PT for AYAs with brain tumors [34], and the cause was suggested to be multifactorial. We could not determine the cause of this negative impact on their daily lives due to the small sample size and the possibility of other aspects being involved. Nevertheless, these insights highlight the fragility of this young population following cancer treatment as they strive to establish a foundation in education, employment, social development, relationships, and family life.

The retrospective design and limited number of patients of our study inherently limit its extrapolation. Additionally, the long period of patient inclusion may have influenced the observed outcomes due to changes in the standard of care and technological advancements. The very low number of patients completing the PEDQOL questionnaire did not allow reporting of long-term QoL outcomes. The use of institutional follow-up questionnaires provides some insights into the impact of treatment and disease, but the findings are based on a nonvalidated tool.

## Conclusion

To our knowledge, this is the first study to report mature clinical outcomes after PBSPT for AYAs with HNS, which are consistent with previous reports on particle therapy. We observed excellent freedom from severe nonocular toxicity at 5 years, and G3 late toxicity tends to develop several years after the treatment, which supports the use of PT as an effective and safe treatment for this young population. Only one patient was treated in a clinical protocol, underlining the barriers that AYAs face for equal access to protocols compared children. Furthermore, a substantial proportion of patients reported a negative impact on their lives due to the disease or the treatments. These data contribute to a better understanding of the challenges of AYAs with sarcomas in the oncology care setting and support the use of PT as a suitable therapeutic modality.
